# Efficacy of smartphone-based retinal photography by undergraduate students in screening and early diagnosing diabetic retinopathy

**DOI:** 10.1186/s40942-022-00388-y

**Published:** 2022-06-07

**Authors:** Jéssica Deponti Gobbi, João Pedro Romero Braga, Moises M. Lucena, Victor C. F. Bellanda, Miguel V. S. Frasson, Daniel Ferraz, Victor Koh, Rodrigo Jorge

**Affiliations:** 1 Division of Ophthalmology, Ribeirão Preto Medical School, University of São Paulo, 3900, Bandeirantes Ave, Ribeirão Preto, SP 14049-900 Brazil; 2 Department of Applied Mathematics and Statistics, University of São Paulo, São Carlos, Brazil; 3 Federal University of São Paulo; D’or Institute of Teaching and Research, São Paulo, Brazil; 4 Department of Ophthalmology, National University Hospital, Singapore, Singapore

**Keywords:** Diabetic retinopathy, Retina, Early diagnosis, Telemedicine, Ophthalmological diagnosis techniques, Low cost technology

## Abstract

**Background:**

To evaluate the efficacy of retinal photography obtained by undergraduate students using a smartphone-based device in screening and early diagnosing diabetic retinopathy (DR)**.**

**Methods:**

We carried out an open prospective study with ninety-nine diabetic patients (194 eyes), who were submitted to an ophthalmological examination in which undergraduate students registered images of the fundus using a smartphone-based device. At the same occasion, an experienced nurse captured fundus photographs from the same patients using a gold standard tabletop camera system (Canon CR-2 Digital Non-Mydriatic Retinal Camera), with a 45º field of view. Two distinct masked specialists evaluated both forms of imaging according to the presence or absence of sings of DR and its markers of severity. We later compared those reports to assess agreement between the two technologies.

**Results:**

Concerning the presence or absence of DR, we found an agreement rate of 84.07% between reports obtained from images of the smartphone-based device and from the regular (tabletop) fundus camera; Kappa: 0.67; Sensitivity: 71.0% (Confidence Interval [CI]: 65.05–78.16%); Specificity: 94.06% (CI: 90.63–97.49%); Accuracy: 84.07%; Positive Predictive Value (PPV): 90.62%; Negative Predictive Value (NPV): 80.51%. As for the classification between proliferative diabetic retinopathy and non-proliferative diabetic retinopathy, we found an agreement of 90.00% between the reports; Kappa: 0.78; Sensitivity: 86.96%; (CI: 79.07–94.85%); Specificity: 91.49% (CI: 84.95–98.03%); Accuracy: 90.00%; PPV: 83.33%; NPV: 93.48%. Regarding the degree of classification of DR, we found an agreement rate of 69.23% between the reports; Kappa: 0.52. As relating to the presence or absence of hard macular exudates, we found an agreement of 84.07% between the reports; Kappa: 0.67; Sensitivity: 71.60% (CI: 65.05–78.16%); Specificity: 94.06% (CI: 90.63–97.49%); Accuracy: 84.07%; PPV: 90.62%; NPV: 80.51%.

**Conclusion:**

The smartphone-based device showed promising accuracy in the detection of DR (84.07%), making it a potential tool in the screening and early diagnosis of DR.

## Background:

Diabetic retinopathy (DR) is one of the most important complications of Diabetes Mellitus (DM) and its incidence is intrinsically related to the duration of the disease and level of glycemic control. [1] Recent reports from the World Health Organization suggest that DR is the cause of visual impairment for 4.2 million people, representing the fifth leading cause of visual impairment and the fourth leading cause of blindness in the world [2]. Early diagnosis of DR allows for intervention that effectively reduces its progression to more severe states [1]. Nevertheless, ophthalmologic follow up for diabetic patients faces severe barriers deriving from the expensiveness of current diagnostic technology and its difficulties of implementation. [3]

Patients with type 1 DM are suggested to undergo ophthalmologic evaluation at puberty or within five years of disease, whereas patients with type 2 DM should be evaluated immediately after being diagnosed. [5] Seven-field stereoscopic photography (gold standard) and ophthalmological examination are admissible methods in the assessment of DR, however, photography shows greater diagnostic sensitivity than clinical examination [6]. Clinical examination in non-specialist settings is usually performed through direct ophthalmoscopy, but its sensitivity is reduced by 50% when performed by clinicians not experienced in detecting DR and without pharmacological mydriasis [6]. As a consequence, telemedicine systems based on digital photographs of the fundus have become increasingly popular, as they allow for assessment of the images by a remotely located ophthalmologist. The diagnostic accuracy of telemedicine using digital images has proven itself to be high and cost-effective in DR screening [3].

In recent years, smartphone adapters for fundus photography have been progressively developed and presented promising results when compared to the reference standards [7][7][7]. Smartphones can be used to register fundus images either serving as slit lamp adapters, as well as integrating direct or monocular indirect ophthalmoscopy settings. [10] In that sense, smartphone-based devices could facilitate earlier detection of DR due to the additional conveniences of portability, easy handling, low cost and the possibility of directly sharing the obtained images with remotely located specialists.

Different professionals are capable of obtaining retinal fundus photographs through smartphone-based methods. Nonetheless, most of the available studies involved the participation of experienced technicians for obtaining the images [7][7][7]. In this study, images of the fundus registered through the smartphone-based device were captured by undergraduate medicine and nursery students who had no previous experience in retinal imaging. Our aim was to assess the method when applied to a realistic scenario, where this technology would be handled by general physicians and nurses with no previous experience in eye imaging, in a context of primary healthcare.

## Materials and methods

### Patients and ethics

We conducted a prospective, open study, collecting data from 116 diabetic patients (231 eyes) at the diabetic retinopathy screening clinic of Hospital das Clínicas de Ribeirão Preto (HC-FMRP-USP), a high complexity general hospital in Brazil. The project was previously approved by the institution’s ethics committee. We included diabetic patients followed up at the hospital who were 18 years old or older and voluntarily agreed to participate in the study. We excluded patients/eyes that presented media opacity, such as cataracts or corneal opacities, and patients who were not able to collaborate with fundus examination, such as those with intense photophobia that could not stay with the eyes open during documentation.

All 116 patients had both eyes examined, except for one who had only one eye. Data from only 97 patients (194 eyes) were included in the study. Thirty-seven eyes were excluded—33 eyes were excluded due to data loss in the HC-FMRP-USP digital medical files system, 3 eyes were excluded due to the presence of cataracts, which prevented the visualization of the fundus, and 1 eye was excluded due to patient photophobia.

### Ophthalmological evaluation

**Fig. 1:**
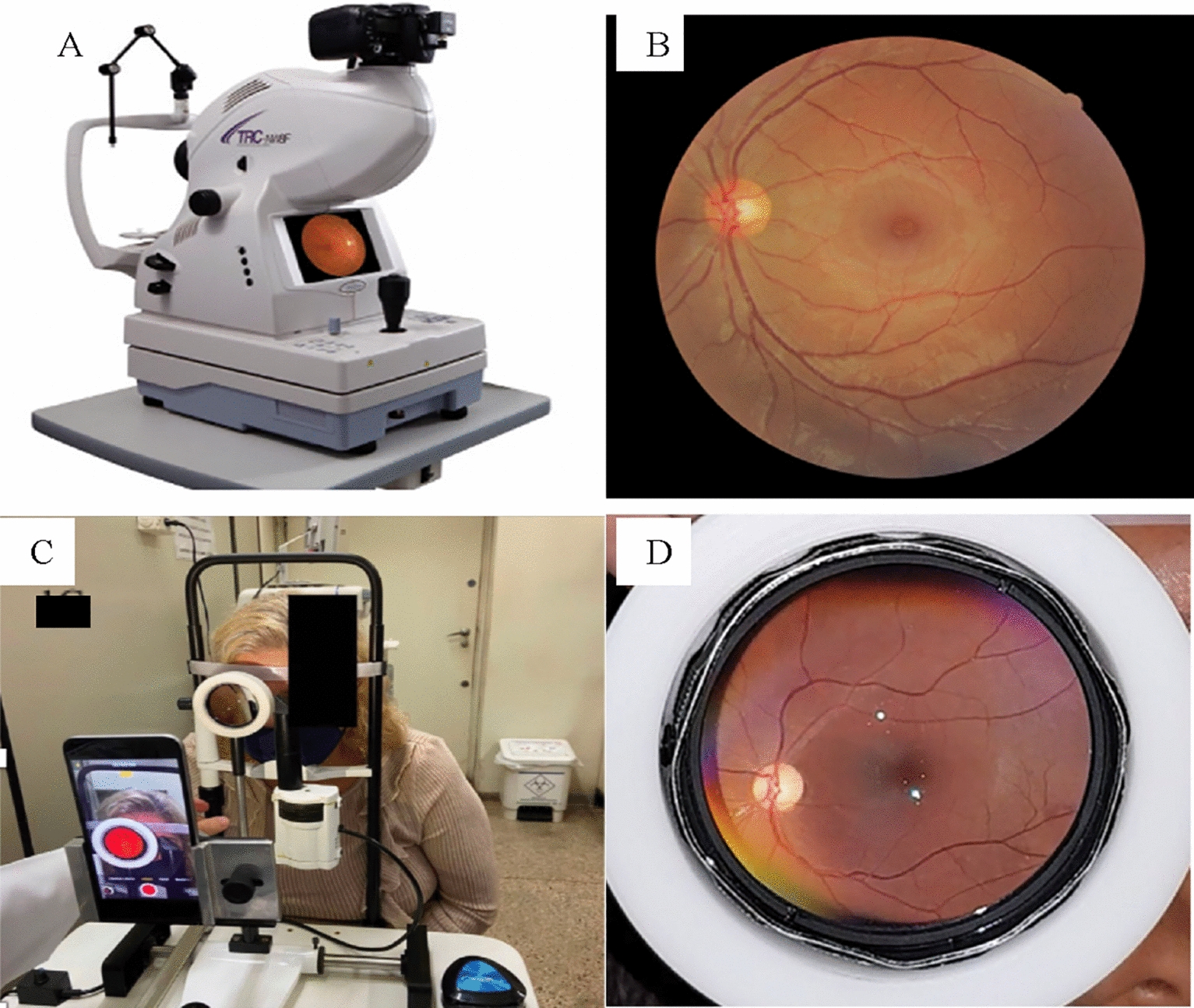
**A** shows the tabletop fundus camera (Canon CR-2 Digital Non-Mydriatic Retinal Camera) and the corresponding color fundus picture of the posterior pole (**B**). **C** shows the smartphone based device used and the corresponding color fundus image captured from the video (**D**). Images do not depict the same patient

**Fig. 2:**
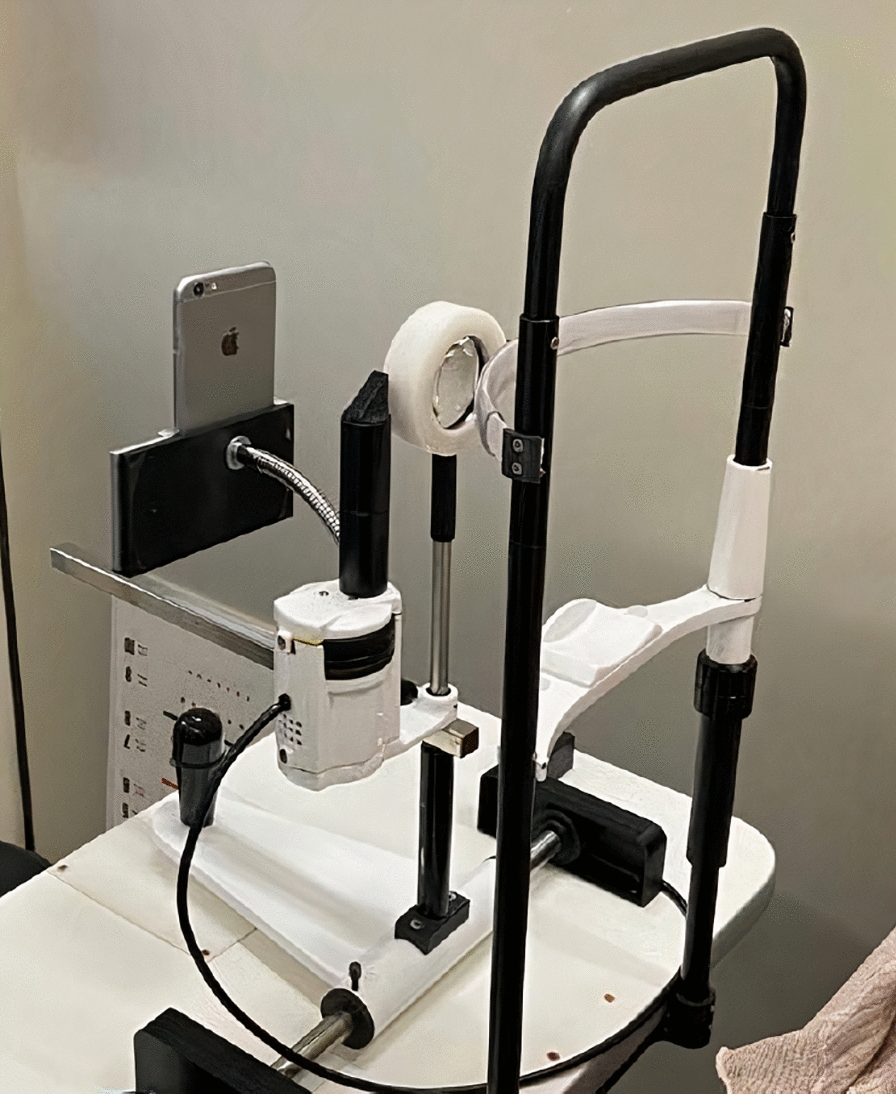
Side view of the smartphone-based device used in the study

During their appointment for diabetic retinopathy evaluation, patients in the study underwent two types of assessments: one being standard seven field color stereoscopic photography of the fundus captured by an experienced nurse through a tabletop fundus camera (Canon CR-2 Digital Non-Mydriatic Retinal Camera—demonstrated on Fig. 1A and B, along with an example of image obtained), and the other being a video documentation of the fundus registered by undergraduate medicine and nursery students through a smartphone-based device (Fig. 1C and D; Fig. 2) shows the exact utilized device and an example of image obtained). Five images were obtained from each eye fundus using the tabletop camera: (1) image centered on the fovea, (2) Temporal retina; (3) Nasal retina; (4) Superior retina; (5) Inferior retina. The undergraduate students who participated in the study were enrolled in the courses of Medicine or Nursery at the Ribeirão Preto Medical School (University of São Paulo) and had no previous experience in eye imaging of any sort.

### Smartphone color fundus documentation

All four participating students received standardized training from an experienced ophthalmologist, who presented the device and explained how to handle it, in addition to monitoring the recording of the first 10 videos. For the smartphone-based examination, the students captured a high-definition video of the fundus, lasting around two minutes each, using a device that consisted of an iron support where a smartphone (in this study, an Apple Iphone 6 ® or a Samsung Galaxy S8 ®) was attached to one side and a 20 D lens was attached to the other side. The device also had an iron adapter on the bottom that allowed its attachment to a slit lamp table. This made image acquisition easier as the patient's head remained fixed by the chin rest, facilitating handling of the camera and adjusting its focus (Fig. 1C and D; Fig. 2). Nothing but the inbuilt camera software of each smartphone were used to register the images. The smartphone’s own flash light was kept on and served as illumination for the entire recording. All the included patients underwent pharmacological mydriasis prior to the exam. After posterior pole focus was obtained, recording was started and the patient was asked to look into five directions in the following order: (1) Straight ahead; (2) Temporally; (3) Nasally; (4) Superiorly and (5) Inferiorly.

### Image analysis by masked retina specialists

Images obtained by each method were saved on cloud storage (Google Drive ®) in a randomized manner and organized by codes. Posteriorly, two independent masked specialists assessed each image individually and classified their findings according to the Airlie-House modified scale [4] (0—Absence of Retinopathy; 1—Minimal non-proliferative diabetic retinopathy [NPDR]; 2—Mild NPDR; 3—Moderate NPDR; 4—Severe NPDR; 5—Very severe NPDR; 6—Proliferative diabetic retinopathy (PDR) with no high risk signs; 7—PDR with high risk signs; 8—Advanced PDR; 9—Classification not possible) and also according to the presence or absence of hard macular exudates, utilized here as a surrogate marker for diabetic macular edema. After each individual analysis, the specialists reported the results in an online form created specifically for that purpose on Google Forms®. Both masked specialists independently evaluated and classified all 194 images generated by the standard fundus camera and then evaluated and classified all 194 videos generated by the smartphone-based method. All images and videos had been completely randomized and identified only by a code, making it impossible for them to identify any patient information. In the same manner, specialist number 1 had no access to the reports produced by specialist number 2 and vice-versa. A third specialist was asked to evaluate cases where there was disagreement between the specialists 1 and 2).

### Statistical analysis

Finally, we calculated the agreement rate, kappa correlation index, sensitivity, specificity and disagreement (false positives and false negatives) of the reports deriving from the smartphone-based method as compared to those deriving from the gold standard tabletop fundus camera system, as well as interobserver agreement between specialists for each method as further detailed ahead. Calculations were performed using the numerical calculation software GNU Octave®.

## Results

### Demographics

Participants had a mean age of 70.5 ± 9.6 years. Self-declared racial demographic was of 73.3% White; 10.1% Black and 16.2% Brown. Enrolled patients had a previous diagnosis of type 1 DM in 45.5% of cases, and of type 2 DM in 54.5% of cases (Table 1).

**Table 1:** Demographic data concerning all 99 patients included in the study

Demographics	
Number of patients	99
Male	40 (40.4%)
Female	59 (56.9%)
Race (self-declared)	
White	73 (73.7%)
Black	10 (10.1%)
Brown	16 (16.2%)
Patients with a previous diagnosis of type 1 DM	45 (45.5%)
Patients with a previous diagnosis of type 2 DM	54 (54.5%)

### Presence or absence of DR

Regarding the presence or absence of DR, agreement between the two independent evaluators of the images (Interobserver) from the smartphone-based device was 88.6% with Kappa of 0.75. As for the gold standard fundus photograph, interobserver agreement was 90.48%, with Kappa of 0.81. Considering reports from the first evaluator (Intraobserver 1), analysis of the smartphone-based device in comparison with the gold standard obtained the agreement of: 82.63%; Kappa: 0.64; Sensitivity: 66.67% (Confidence Interval—CI: 59.96–73.37%); Specificity: 95.28% (CI: 92.27–98.30%); Accuracy: 82.63%; Positive predictive value: 91.80%; Negative predictive value: 78.29%. Considering reports from the second evaluator (Intraobserver 2), smartphone-based device compared to the gold standard showed an agreement of 79.69%; Kappa: 0.60; Sensitivity: 71.29% (CI: 64.89% -77.69%); Specificity: 89.01% (CI: 84.59%—93.43%); Accuracy: 79.69%; Positive predictive value: 87.80%; Negative predictive value: 73.64%. These data are depicted in Tables 2 and 3.

**Table 2:** Frequency of diagnoses comparing the degree of retinopathy as determined by the smartphone-based device and the gold standard

Severity of DR (smartphone-based device)										
	Absent RD	Minimal NPDR	Mild NPDR	Moderate NPDR	Severe NPDR	Very severe NPDR	PDR without signs of high risk	PDR with signs of high risk	Advanced PDR	TOTAL
Absent RD	84	16	14	2	0	0	0	0	0	116
Minimal NPDR	1	0	2	0	0	0	0	0	0	3
Mild NPDR	3	0	11	2	1	0	2	0	0	19
Moderate NPDR	1	0	3	7	2	0	1	0	0	14
Severe NPDR	0	0	0	0	4	0	0	1	0	5
Very severe NPDR	0	0	0	0	0	0	0	1	0	1
PDR without signs of high risk	0	0	0	1	0	0	16	0	0	17
PDR with signs of high risk	0	0	0	0	0	0	2	2	1	5
Advanced PDR	0	0	0	0	0	0	0	0	2	2
TOTAL	89	16	30	12	7	0	21	4	3	182

*DR* Diabetic retinopathy, *NPDR* non-proliferative diabetic retinopathy, *PDR* proliferative diabetic retinopathy

**Table 3:** Values of interobserver and intraobserver agreement when the presence or absence of DR

	Agreement on the presence or absence of diabetic retinopathy
Interobserver Agreement (smartphone based device)	88.6% (Kappa 0.75)
Interobserver Agreement (gold standard)	90.48% ( Kappa 0.81)
Intraobserver Agreement 1( device x gold standard)	82.63% (Kappa 0,64)
Intraobserver Agreement 2 (device x gold standard)	79.69% (Kappa 0,60)

### Proliferative vs non-proliferative DR

Concerning the classification between proliferative diabetic retinopathy and non-proliferative diabetic retinopathy, interobserver agreement of the images from the smartphone-based device was 94.83%, with Kappa of 0.89; and in the gold standard images the interobserver agreement was 92, 50%, with Kappa of 0.83. Intraobserver 1: smartphone-based device analysis compared to gold standard images demonstrated agreement: 89.47%; Kappa: 0.78; Sensitivity: 93.94% (CI: 97.74–100.13%); Specificity: 83.33% (CI: 73.66–93.01%); Accuracy: 89.47%; Positive predictive value: 88.57%; Negative predictive value: 90.91%. Intraobserver 2: analysis of the smartphone-based device in comparison with the gold standard images showed agreement: 90.72%; Kappa: 0.81; Sensitivity: 94.44% (CI: 88.83–100.06%); Specificity: 85.71% (CI: 77.14–94.29%); Accuracy: 90.62%; Positive predictive value: 89.47%; Negative predictive value: 92.31%. These data are shown in Tables 2 and 4.

**Table 4:** Interobserver and intraobserver agreement values for the presence of proliferative or non-proliferative DR

	Agreement in classification between proliferative and non-proliferative diabetic retinopathy
Interobserver Agreement (smartphone based device)	94.83% ( Kappa 0.89)
Interobserver Agreement (gold standard)	92.50% (Kappa 0.83)
Intraobserver Agreement 1 (device x gold standard)	89.47% ( Kappa 0.78)
Intraobserver Agreement 2 (device x gold standard)	79.69% ( Kappa 0.60)

### Classification of severity

For the analysis of the classification of severity of DR, when specialists differed by only one class, we considered only the most severe classification. In this case, interobserver agreement found in the images of the smartphone-based device was 83.94%, and Kappa: 0.76. In the gold standard images, interobserver agreement was 90.67%, and Kappa: 0.87. Intraobserver 1: agreement of the reports obtained by the smartphone-based images in comparison with those coming from the gold standard was 86.01% and Kappa: 0.77. Intraobserver 2: agreement of the reports obtained by the smartphone-based images in comparison with those coming from the gold standard was 87.56% and Kappa: 0.82.

Considering a tolerance of up to two classes of divergence, agreement found in the interobserver comparison of the images obtained by the smartphone-based device was 93.78%, and Kappa: 0.90. Interobserver comparison of the images obtained by the gold standard was 94.30%, and Kappa: 0.92. Intraobserver 1: agreement of the reports obtained by the smartphone-based images in comparison with those coming from the gold standard was 97.93%, and Kappa: 0.97. Intraobserver 2: agreement of the reports obtained by the smartphone-based images in comparison with those coming from the gold standard was 97.41%, and Kappa: 0.96.

### Hard macular exudates

Considering the presence or absence of hard macular exudates, agreement of the reports obtained by the smartphone-based images in comparison with those coming from the gold standard was 84.07%, with Kappa of: 0.67; Sensitivity: 71.60% (confidence interval—CI: 65.05–78.16%); Specificity: 94.06% (confidence interval—CI: 90.63–97.49%); Accuracy: 84.07%; Positive predictive value: 90.62%; Negative predictive value: 80.51%.

### Final analysis

In order to obtain a final analysis between the two methods, results from the two specialists were merged. On reports from both the smartphone-based and the conventional tabletop camera methods, when the classification attributed by the specialists was consensual in their analysis, the data was kept; when there was no consensus, a third independent masked specialist assessed and assigned the final analysis. With this approach, the number of included eyes dropped to 182, as the third specialist classified 12 eyes that were not in consensus among the first specialists as “not possible to classify”, and they were excluded from the final analysis.

Therefore, taking into account the result from the consensus obtained, in relation to the presence or absence of DR, the final agreement between the images of the two methods was 84,07%, with Kappa of 0.67; Sensitivity: 71.0% (confidence interval—CI: 65.05–78.16%); Specificity: 94.06% (confidence interval—CI: 90.63–97.49%); Accuracy: 84.07%; Positive predictive value: 90.62%; Negative predictive value: 80.51%.

As for the classification between proliferative diabetic retinopathy and nonproliferative diabetic retinopathy, final agreement between the images from the smartphone-based device and those from the gold standard was 90.00%; with Kappa of: 0.78; Sensitivity: 86.96%; (confidence interval—CI: 79.07–94.85%); Specificity: 91.49% (confidence interval—CI: 84.95–98.03%); Accuracy: 90.00%; Positive predictive value: 83.33%; Negative predictive value: 93.48%.

Regarding the classification of severity of DR, to obtain a final result, when the specialists differed by only 1 class, the most severe classification was assigned, when they differed by up to 2 classes, a third independent masked specialist performed the analysis and attributed the final classification (Tables 2 and 5). Therefore, agreement of the reports obtained by the smartphone-based images in comparison with those coming from the gold standard was 69.23% with Kappa of: 0.52.

**Table 5:** Sensitivity and specificity of smartphone-based device ocular fundus images according to diabetic retinopathy severity scale

	Sensibility (95% CI)	Specificity (95% CI)
Absent RD	0.94 (0.87–0.98)	0.66 (0.55–0.75)
Minimal NPDR	0.00 (0.01–0.24)	0.98 (0.94–1.00)
Mild NPDR	0.37 (0.21–0.56)	0.95 (0.90–0.98)
Moderate NPDR	0.58 (0.29–0.84)	0.96 (0.91–0.98)
Severe NPDR	0.57 (0.20–0.89)	0.99 (0.96–1.00)
Very severe NPDR	*	0.99 (0.97–1.00)
PDR without signs of high risk	0.76 (0.52–0.91)	0.99 (0.96–1.00)
PDR with signs of high risk	0.50 (0.09–0.92)	0.98 (0.95–1.00)
Advanced PDR	0.67 (0.13–1.00)	1.00 (0.97–1.00)

*DR* diabetic retinopathy, *CI* confidence interval, *NPDR* non-proliferative diabetic retinopathy, *PDR* proliferative diabetic retinopathy

^*^There was no diagnosis of very severe NPDR by the gold standard method, so there is no calculation for sensitivity

## Discussion

Our study was able to verify that retinal images obtained by undergraduate students using a smartphone-based device showed satisfactory performance when compared to the reference standard for the diagnosis of DR.

Recent studies suggest that the diagnostic accuracy of telemedicine using digital images in DR is, in general, high. Sensitivity of telemedicine exceeded 80% in detecting the absence of DR, low- or high-risk proliferative diabetic retinopathy (PDR), and exceeded 70% in detecting mild or moderate non-proliferative diabetic retinopathy (NPDR) [3].The high sensitivity of its detection of any clinical level of DR indicates that telemedicine could be widely used for DR screening [3]. Portable devices for eye fundus image acquisition have shown high levels of agreement with traditional tabletop retinal cameras for the detection and follow-up of DR [7]. However, the latter tend to perform better compared to smartphone-based devices like the one reported in this study. Russo et al. [8] compared biomicroscopy to a device (D-EYE®) that turns the smartphone into a portable fundus camera by using its own constitutional camera and LED light. The study reported substantial agreement between the methods, with sensitivity and specificity of 0.89 and 1.0, respectively, to detect proliferative DR; and of 0.89 and 1.0, respectively, to detect macular edema. Toy et al. [9], evaluated the photographs obtained by a smartphone-based device (Paxos Scope®), attached to a 20D lens, in comparison with clinical examination, finding good agreement, with a sensitivity of 91% and a specificity of 99% for the detection of DR. In the same study, the authors recommended that it would be interesting to compare a smartphone-based device with a tabletop fundus camera, the gold standard for diagnosing DR.

In the present study, we found a sensitivity of 0.71 and a specificity of 0.94 to detect the presence of DR at any level; a sensitivity of 0.76 and a specificity of 0.99 to detect proliferative DR; as well as a sensitivity of 0.72 and a specificity of 0.94 to detect macular exudates. We attribute the lower values of sensitivity and specificity in the present study to the fact that the users of the smartphone-based fundus camera were not used to fundus photography, while in the previous studies smartphone-based ophthalmoscopy was performed by a retina specialist [8, 9]. In their study, Williams et al. stated that there is level I evidence that single-field fundus photography with interpretation by trained readers can serve as a screening tool to identify patients with diabetic retinopathy for referral for ophthalmologic evaluation and treatment, but it is not a substitute for a comprehensive eye examination [11]. Ryan ME et al. reported that photographs from smartphones assisted by 20 diopters lenses had a low rate of unclassifiable images, and most of them had at least satisfactory quality. The sensitivity and specificity of smartphone photographic detection of DR compared with the conventional photographs were 50% (95% CI, 43–56) and 94% (95% CI, 92–97), respectively. Kappa was 0.48 (95% CI, 0.41–0.56), indicating moderate agreement between the smartphone and the 7-field mydriatic photographs. Our study, regarding the presence or absence of DR, showed a Kappa of 0.67, sensitivity of 71.0% (confidence interval—CI: 65.05–78.16%) and specificity of 94.06% (CI: 90.63–97.49%). The smartphone was less sensitive than non-mydriatic photography in detecting the presence of DR at any degree. However, the two methods were similar in detecting vision threatening stages of the disease. Although both methods have shown robust specificity, smartphone-based teleophthalmology screening represents a much lower cost of implementation, and could be particularly useful as a tool that allows for detection of the disease in patients who may not have proper access to eye care [12]. Furthermore, considering that artificial intelligence (AI) systems are currently being developed and gradually implanted worldwide [13, 14], it is plausible to assume that the portability of smartphone-generated images could, in a near future, act synergistically with the power of AI in order to amplify access to eye care.

In line with the other studies in literature (Russo et al. and Toy et al.), our study confirmed two important aspects of screening for DR through a smartphone-based fundus camera: its specificity tends to be greater than its sensitivity, and its sensitivity is always increased for the detection of the proliferative phase of the disease, where findings are more exuberant when compared to the initial stages, which present with only discrete microaneurysms and microhemorrhages.

## Conclusion

High cost and low availability of eye examination, especially when requiring in-site experts, represent an important limitation for DR screening. Fundus images taken through a smartphone-based method by undergraduate students, here adopted as surrogates for professionals with no previous experience in eye imaging, may favor early diagnosis and severity classification of DR. Implementation of this method in primary healthcare settings (such as the basic care units of Brazil's public health system) could allow for broader detection and timely referral for intervention in a large population of underserved diabetic patients.

## Data Availability

All data generated in this study, including the images obtained through both the analysed method and the gold standard, were saved on private cloud storage (Google Drive ®) for patient safety and privacy. We kindly request any interested parts to contact the authors directly for obtaining access to the database when applicable.

## Abbreviations

DR: Diabetic retinopathy
DM: Diabetes mellitus
CI: Confidence Interval
NPDR: Non proliferative diabetic retinopathy
PDR: Proliferative diabetic retinopathy

## References

[1] Klein R, Klein BEK, Flynn HW, Smiddy WE (2000). Epidemiology of eye disease in diabetes. Diabetes and ocular Disease: past, present, and future therapies.

[2] World Health Organization (2015). Tool for the assessment of diabetic retinopathy and diabetes management systems.

[3] Shi L, Wu H, Dong J, Jiang K, Lu X, Shi J (2015). Telemedicine for detecting diabetic retinopathy: a systematic review and meta-analysis. Br J Ophthalmol.

[4] Early Treatment Diabetic Retinopathy Study Research Group (1991). Grading diabetic retinopathy from stereoscopic color fundus photographs–an extension of the modified Airlie House classification. ETDRS report number 10. Ophthalmology.

[5] Sociedade Brasileira de Diabetes. Diretrizes da Sociedade Brasileira de Diabetes (2015–2016). São Paulo, SP: A.C. Farmacêutica, 2016.

[6] Fong S, Aiello LP, Gardner TW, King GL (2003). Diabetic retinopathy. Diabetes Care.

[7] Hilgert GR, Trevizan E, de Souza JM (2019). Uso de retinógrafo portátil como ferramenta no rastreamento de retinopatia diabética. Rev Bras Oftalmol.

[8] Russo A, Morescalchi F, Costagliola C, Delcassi L, Semeraro F (2015). Comparison of smartphone ophthalmoscopy with slit-lamp biomicroscopy for grading diabetic retinopathy. Am J Ophthalmol.

[9] Toy BC, Myung DJ, He L (2016). Smartphone-based dilated fundus photography and near visual acuity testing as inexpensive screening tools to detect referral warranted diabetic eye disease. Retina.

[10] Bolster NM, Giardini ME, Bastawrous A (2016). The diabetic retinopathy screening workflow: potential for smartphone imaging. J Diabetes Sci Technol.

[11] Williams GA, Scott IU, Haller JA, Maguire AM, Marcus D, McDonald HR (2004). Single-field fundus photography for diabetic retinopathy screening. Ophthalmology.

[12] Ryan ME, Rajalakshmi R, Prathiba V, Anjana RM, Ranjani H, Narayan KMV (2015). Comparison among methods of retinopathy assessment (CAMRA) study. Ophthalmology.

[13] Abràmoff MD, Lavin PT, Birch M (2018). Pivotal trial of an autonomous AI-based diagnostic system for detection of diabetic retinopathy in primary care offices. NPJ Digit Med.

[14] Vedula SS, Tsou BC, Sikder S (2022). Artificial intelligence in clinical practice is here—now what?. JAMA Ophthalmol.

